# Thyroxine in acute myocardial infarction (ThyrAMI) - levothyroxine in subclinical hypothyroidism post-acute myocardial infarction: study protocol for a randomised controlled trial

**DOI:** 10.1186/s13063-015-0621-5

**Published:** 2015-03-25

**Authors:** Avais Jabbar, Lorna Ingoe, Simon Pearce, Azfar Zaman, Salman Razvi

**Affiliations:** Institute of Genetic Medicine, Newcastle University, Central Parkway, Newcastle upon Tyne, NE1 3BZ UK; Department of Endocrinology, Gateshead Health NHS Foundation trust, Sheriff Hill, Gateshead, NE9 6SX UK; Endocrine Unit, Newcastle upon Tyne Hospitals NHS foundation trust, Queen Victoria Road, Newcastle upon Tyne, NE1 4LP UK; Department of Cardiology, Newcastle upon Tyne Hospitals NHS Foundation Trust, Freeman Road, NE7 7DN Newcastle upon Tyne, UK; Institute of Cellular Medicine, Newcastle University, Newcastle upon Tyne, NE2 4HH UK; Newcastle University, Queen Elizabeth Hospital, Sheriff Hill, Gateshead, NE9 6SX UK

## Abstract

**Background:**

Cardiac disease is the most common cause of morbidity and mortality in the United Kingdom. Even minor changes in thyroid hormone concentration may impact adversely on the cardiovascular system. Subclinical hypothyroidism (SCH) after admission for an acute cardiac problem has been associated with an increase in cardiac mortality and overall death. We have designed protocols for a prospective observational study to assess the association of thyroid function at the time of acute myocardial infarction (AMI) with cardiovascular outcomes, and a double-blinded randomised placebo-controlled trial of levothyroxine to evaluate its effect on LV function and vascular health.

**Methods/Design:**

ThyrAMI 1: This will be a prospective longitudinal observational study of patients with AMI that will be followed for 24 months to study the association between thyroid status at the time of AMI (within 24 hours of diagnosis) with vascular outcomes.

ThyrAMI 2: This will be a prospective double-blinded randomised placebo-controlled trial of levothyroxine of 12 months duration in patients with AMI and SCH.

Setting: Patients will be recruited from five hospitals in the North East of England.

Participants: One hundred patients with thyroid function tests within the subclinical hypothyroid range upon admission with an AMI and no previous history of thyroid disease.

Intervention: Levothyroxine will be administered at a starting dose of 25 mcg once daily, which will be increased at intervals if needed to maintain a TSH level between 0.4 to 2.5 mU/L, versus a placebo.

Randomisation: Participants will be randomized with a computerised randomisation algorithm, stratified by type of MI (NSTEMI versus STEMI), in a 1:1 ratio to levothyroxine therapy or placebo (as container or bottle numbers), starting within 21 (+/− 7) days of AMI.

Blinding: Assignment to either the LT4 or placebo arm will be double-blinded.

Outcomes: The outcome will be the effect of levothyroxine on ventricular function, endothelial function and blood coagulability and rheology.

**Discussion:**

There is evidence to suggest that treatment of SCH can improve cardiovascular parameters. Therefore, ThyrAMI 1 and ThyrAMI 2 will be the first trials investigating SCH in AMI to give a better insight into whether thyroid hormone levels are a key target for improving cardiovascular outcomes.

**Trial registration:**

ISRCTN number: ISRCTN52505169. Date of registration: 09/01/2015

## Background

Cardiac disease is the most common cause of morbidity and mortality in the United Kingdom, costing £29 billion [1]. Much research on cardiovascular disease has focused on men, as these diseases occur at younger ages and are more severe in males, and risk factors for cardiovascular disease in women are relatively understudied. Thyroid disease is predominantly a disease of women and is increasingly being recognised as an adverse cardiac risk factor. Levothyroxine is cheap (costs 5 pence/day), safe and commonly used generic drug (prescribed to 3% of the UK population). The appropriate use of levothyroxine in SCH patients with and without heart disease has the potential to optimise management of hypothyroidism to minimise vascular risk and to improve outcomes of AMI. The myocardium and vascular endothelial tissues have receptors for thyroid hormones and are sensitive to changes in circulating thyroid hormone concentration [2]. Even minor changes in thyroid hormone concentration may impact adversely on the cardiovascular system. Importantly, both mild hypothyroidism (SCH) and hyperthyroidism have been associated with a 20 to 80% increase in vascular morbidity and mortality risk [3-7]. This has huge population significance as a vascular risk factor, since at least 10% of older women have SCH, but this is under-recognised, owing possibly to a lack of commercial exploitability. Small intervention trials of levothyroxine in SCH have shown improvement in left ventricular function, vascular endothelial function and atherogenic lipid particles [8]. SCH after an acute cardiac problem has been associated with an up to 3.6-fold increase in cardiac mortality and a 2.3-fold increase in overall death [9,10]. Following experimental coronary artery ligation in an animal model of AMI, heart failure is associated with reduced thyroid hormone receptor expression in the myocardium, leading to tissue hypothyroidism [11]. Moreover, thyroid hormone administration improves cardiac contractility, left ventricular (LV) function, and augments myocardial remodelling [12]. In addition, thyroid hormone regulates angiogenesis, cardio-protection, cardiac metabolism, and myocyte regeneration at a molecular level, changes that can reverse left ventricular remodelling by favourably improving myocyte shape and geometry of LV cavity, thus improving recovery from AMI [13].

Analysis of more than 3,000 participants aged 65 years or older without heart failure at baseline, who were followed up for 12 years in the Cardiovascular Health Study, showed that SCH participants who were treated with levothyroxine had a 72% reduction in heart failure events [14]. Several other studies have shown similar results [15-17]. Thus, even mild hypothyroidism following AMI could be an important marker for poor outcome, and one that is ripe for cost-effective intervention if level 1 evidence of efficacy can be shown. In a recent preliminary analysis, we have shown that thrombus area, as measured by a model that simulates *ex vivo* coronary artery blood flow, is higher in people with SCH 7 to 10 days post non-ST elevation AMI despite dual anti-platelet therapy (with aspirin and clopidogrel) [18]. In multivariate analysis, serum TSH concentrations were directly associated with thrombus area, with a strength of association similar to other established cardiac risk factors such as diabetes mellitus and hypertension [18]. It is currently unknown whether levothyroxine treatment in SCH patients after an AMI can improve ventricular function, improve endothelial function and improve blood coagulability and rheology. Therefore, this project aims to provide level 1 evidence to answer these important questions. This programme has two separate yet interlinked studies: ThyrAMI 1 - an observational longitudinal study to assess association of thyroid function at the time of AMI with cardiovascular outcomes, and ThyrAMI 2 - a randomised placebo-controlled interventional study of levothyroxine in SCH patients to evaluate effect on LV function and vascular health.

## Methods/Design

### ThyrAMI 1

#### Aim

The aim of this trial is to assess what proportion of individuals with AMI have thyroid dysfunction and whether thyroid function at the time of AMI is related to adverse vascular outcomes.

### Study design

**Figure 1:**
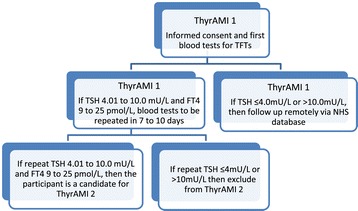
**Flow chart representing ThyrAMI 1 observational study and identification of potential cohort for the interventional trial ThyrAMI 2.**

The study is designed as a prospective longitudinal observational study of unselected patients with AMI who will be followed for 24 months (Figure 1).

### Primary objectives

The primary objective is to assess the association between thyroid status at the time of AMI (within 24 hours of diagnosis) with vascular outcomes (another AMI, coronary revascularisation or death due to ischaemic heart disease) over 24 months.

### Secondary objectives

The secondary objectives are as follows:

1. The proportion of individuals with thyroid dysfunction at the time of AMI (overt hypo- and hyperthyroidism, subclinical hypo- and hyperthyroidism, and isolated low FT3)
2. Association between thyroid function at the time of AMI with markers of severity of AMI (troponin, LV function, and coronary artery disease on angiogram).
3. Relation between thyroid function at the time of AMI with all-cause mortality over 24 months.
4. Association between thyroid function at the time of AMI with new diagnoses of heart failure, atrial fibrillation or stroke.

### Participant population

The study population are patients post-AMI in five different hospitals in the North East of England.

Inclusion criteria are as follows:

1. Adult males and females (aged 18 years or older).
2. Acute myocardial infarction diagnosed in the preceding 24 hours (defined as chest pain with dynamic ECG changes or increased troponin enzymes (at least a fourfold increase above the local laboratory reference range).
3. Participants in other research studies will be eligible for inclusion as this is an observational study.

Exclusion criteria are as follows:

1. Patients who are unable to provide informed consent.
2. Those with advanced malignancy (who are unlikely to survive for more than 6 months in the opinion of local investigator).
3. Those on medications that can affect thyroid function such as amiodarone, lithium, carbimazole and propylthiouracil. Patients on levothyroxine will be included, but their results will be analysed separately.

### Screening, recruitment and consent

#### Identification, screening and recruitment of participants

Eligible participants will be identified from acute hospitals. All individuals that fit the study criteria will be approached and provided with a participant information sheet for ThyrAMI 1, and informed consent will be obtained. They will have their thyroid function checked, and a case record form will be completed. Patients that are otherwise eligible to be included but have been discharged and have not had their thyroid function checked will be approached via telephone, and initial consent will be obtained prior to requesting these tests be done on the stored serum, if still available in the Laboratory. A screening log will be kept at each site with a unique identifier for each participant who is approached for the study. Recruitment will take place over a period of 12 months in all participating centres See Figure 1.

### Consent procedures

All informed consent discussions will be undertaken by experienced research staff (per delegation log) involved in the study with an opportunity for participants to ask any questions. Following receipt of information about the study (that is, provision of the study invitation pack), participants will be given reasonable time to decide whether or not they would like to participate. Those wishing to take part will provide written informed consent by signing and dating the study consent form and initialling relevant sections, which will be witnessed and dated by a member of the research team with documented, delegated responsibility to do so. Participants for ThyrAMI 1 will be informed via the Participant Information Sheet that they may be approached to consider participating in ThyrAMI 2 if their thyroid blood tests are in the inclusion range.

The original signed consent form will be retained in the Investigator Site File, with a copy in the relevant clinical notes and a copy provided to the participant. The participant will specifically consent to their GP being informed of their participation in the study. The right to refuse to participate without giving reasons will be respected.

The information sheet and consent form for the study will be available only in English. Interpreters will be arranged for all visits of participants who require them either for verbal translation or for deaf participants wishing to take part in the study, via local NHS arrangements. Qualified interpreters will be used to explain the consent form and information sheet, and great priority will be placed on finding the most direct communication.

### Data analysis

The relationship between the outcomes and thyroid status categories (euthyroid, subclinical hypothyroid, subclinical hyperthyroid and isolated low FT3) will be examined. A chi-squared test will be used to investigate the association between categorical measures of thyroid status and categorical and/or binary measures of CV outcome. Independent sample t-tests will be used to investigate the association between continuous measures of thyroid function (TSH, FT4 and FT3) and binary measures of CV outcome. Cox regression models will be used to explore the association between continuous and categorical measures of CV with survival (time to event).

### ThyrAMI 2

#### Objective

To determine whether treatment of SCH with levothyroxine following AMI improves left ventricular function, thrombus area, endothelial function, health status, quality of life and is safe.

### Study design

This study is a prospective randomised placebo-controlled trial of levothyroxine of 12 months duration in patients with subclinical hypothyroidism (serum TSH persistently 4.01 to 10.0 mU/l, normal FT4) starting within 21 days following AMI. The day of AMI is the date of diagnosis or the date of admission to hospital, whichever is later.

### Primary outcome measure

The primary outcome measure will be change in LV ejection fraction as assessed by magnetic resonance imaging. Cardiac magnetic resonance (MR) is considered the gold standard for evaluating cardiac volumes and function [19]. Gradient echo sequences provide a naturally high level of contrast between intracavitary blood and myocardium, thus allowing an accurate and reproducible determination of LV volumes, mass and calculation of stroke volume and ejection fraction. In this context, additional information can also be drawn from application of the more recent cardiac MR tagging analysis, which represents a noninvasive highly accurate method for direct quantification of regional systolic function [20]. Cardiac MR imaging will be performed in the dedicated 3 T MRI centre at Newcastle University.

### Secondary outcome measures

Secondary outcome measures will include the following:

1. Left ventricular systolic and end diastolic volumes and myocardial viability: Cardiac MR parameters of both global and segmental LV function measurements will be obtained as per standardised protocols already in place in the Newcastle MR centre using a steady state free precession or fast gradient echo technique. In addition, further scans will be obtained 15 to 20 minutes after intravenous gadolinium-chelate administration (delayed enhancement MRI). Regions of nonviable myocardial tissue will be identified as areas of increased enhancement (hyper enhancement) whereas viable tissue will have null (black) on the acquired images.
2. Thrombus burden: The Badimon chamber is a highly reproducible clinical *ex vivo* model of thrombosis that mimics flow conditions within the coronary circulation of man [21]. The device incorporates two chambers with internal flow channels of different diameters, one simulating high-shear flow of 1690 ml/s and the other simulating low-shear flow. The internal flow channels are lined with porcine aortic tunica media - the thrombogenic substrate. After perfusion with venous blood from the patient, flowing at 10 mL min −1 for 5 min, aortic segments are fixed in 10% formalin for 72 h. Total thrombus burden is measured using validated computer-assisted planimetry using IMAGE-PRO PLUS software (Media Cybernetics, Inc., Bethesda, MD, USA). The results are the mean of the analysed sections (μm2 .mm-1). The measurements will be performed at the Clinical Research Facility (CRF), where there are professionals who have considerable experience using this technique in other high vascular risk groups.
3. Thromboelastography (TEG™): This is a noninvasive measure for evaluating efficiency and quality of *ex vivo* thrombus formation. Quantitative measures include clotting time parameters, clot strength and clot lysis. These parameters have been validated as highly correlated with longer term cardiac outcomes in several conditions of risk [22,23]. TEG™ will be measured at the CRF, RVI, Newcastle.
4. Endothelial function: Endothelial dysfunction is the earliest stage in the atherosclerosis disease process. In this study, endothelial function will be assessed by measuring peripheral arterial tone using a validated tool, EndoPAT™ [24]. Endothelial function assessment using the EndoPAT™ has been shown to have a high degree of correlation with coronary artery endothelial function [25], the severity and extent of coronary artery disease [26], and traditional cardiovascular risk factors [27] and is useful in predicting future cardiovascular events [28]. EndoPAT™ assessments will be performed at the Clinical Research Facility, RVI, Newcastle at baseline and at the end of the study.
5. Platelet reactivity: The reactivity (inhibition) of platelets to anti-platelet agents such as aspirin, clopidogrel, prasugrel and ticagrelor will be quantified by the point-of-care monitor VerifyNow™ (Accumetrics, CA, USA). Arachidonic acid reactive units and P2Y12 reactive units will be recorded at baseline and at the end of the study. This test will not be carried out in individuals who take anticoagulants such as warfarin.
6. Safety assessments: The safety of levothyroxine therapy in post-AMI patients will be assessed at each study visit by enquiry of symptoms by New York Heart Assessment (NYHA) category classification, ECG recording for rhythm disturbance and peripheral oxygen levels by pulse oximetry (at rest).
7. QoL and depression: This will be assessed by validated tool of measuring health status, the Short Form 12 four week recall (SF-12™), a disease specific questionnaire for heart failure, the Minnesota Living With Heart Failure Questionnaire™, and the Centre for Epidemiologic Studies Depression Scale (CES-D), at baseline and at the end of the study.

### Participant population

#### Inclusion criteria

The inclusion criteria are as follows:

1. Males and females aged between 18 to 75 yrs.
2. Serum TSH between 4.01 to 10.00 mU/L with normal free thyroxine levels (9 to 25 pmol/L) on two occasions (on day of admission for AMI and 7 to 10 days after AMI).
3. Acute myocardial infarction diagnosed on admission to hospital (chest pain with dynamic ECG changes or increase troponin enzymes (at least a fourfold increase above the laboratory reference range).

### Exclusion criteria

The exclusion criteria are as follows:

1. Patients on medications that affect thyroid function (levothyroxine, carbimazole, propylthiouracil, amiodarone, lithium).
2. Patients who are unable to provide written informed consent.
3. Patients with advanced malignancy (who, in the opinion of the investigator, are unlikely to survive for more than 6 months).
4. Patients with sustained ventricular tachycardia requiring treatment that occurs >24 hrs after myocardial reperfusion/revascularisation.
5. Patients who have contra-indications to MR scanning (cardiac pacemaker, metallic heart valves, cochlear implants, coronary artery stents incompatible with MR scanning, *etcetera*).
6. Patients who are unlikely or unwilling, in the opinion of the investigator, to attend for study-specific visits.
7. Participants whose serum TSH is >10.0 or <4.0 on either occasion.
8. Patients who are already participating in another interventional study.

### Screening, recruitment and consent

#### Identification, screening and recruitment of participants

Eligible participants will be identified from the cardiology units of acute hospitals in the North East of England (Gateshead, Newcastle, Sunderland, South Tyneside and North Tyneside Hospitals). All individuals who fit the study inclusion and exclusion criteria will have their thyroid function checked, after informed consent is obtained, within 24 hours of diagnosis of AMI. Of these patients, those that have TSH and FT4 levels within the inclusion range will be provided with a participant information sheet for ThyrAMI 2. Furthermore, these individuals will be invited to have a repeat thyroid blood test 7 to 10 days after the day of AMI. The day of AMI is the date of diagnosis or the date of admission to the hospital, whichever is later. Those individuals who have TSH and FT4 levels within the inclusion range on the second occasion will be asked to participate in the study and sign the consent form (Figure 1). Those who do not attend for their second visit blood test will be contacted to enquire whether they are still interested in participating.

**Figure 2:**
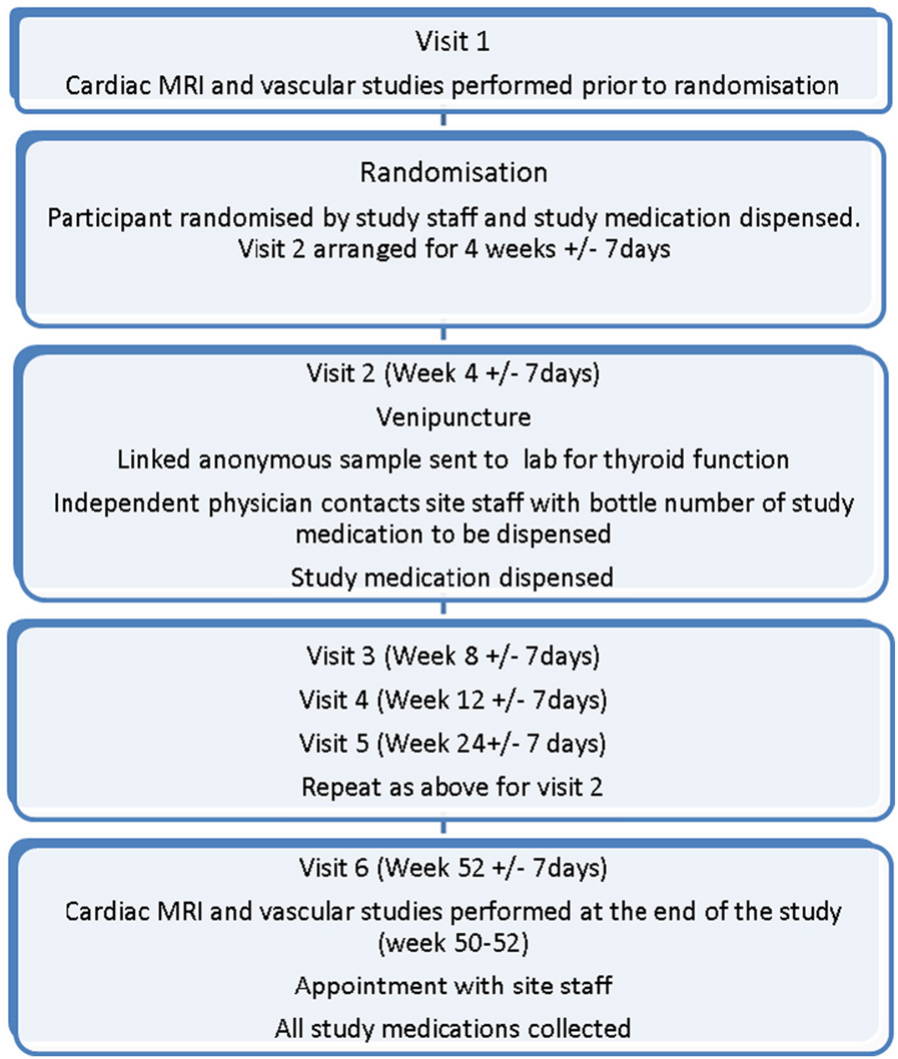
**Flow chart representing the levothyroxine post-acute myocardial infarction in patients with subclinical hypothyroidism interventional trial (ThyrAMI 2).**

Once a participant has had thyroid blood results that are within the desired range for inclusion in the study on two occasions and has consented to participate in the study, the study team will arrange a convenient date and time for the baseline tests and randomisation to the treatment groups (see Figure 2). The dates for the baseline tests will be within 21 days of the patients’ AMI dates.

A screening and recruitment log will be held securely at the Investigator sites. The screening log will document anonymised details of patients invited to participate in the study. A participant recruitment/ID log will record those who consented to participate in the trial.

### Consent procedures

All informed consent discussions will be undertaken by experienced research staff (per delegation log) involved in the study with an opportunity for participants to ask any questions. Following receipt of information about the study (that is, provision of the study invitation pack), participants will be given reasonable time to decide whether or not they would like to participate. Those wishing to take part will provide written informed consent by signing and dating the study consent form and initialling the relevant sections, which will be witnessed and dated by a member of the research team with documented, delegated responsibility to do so. Written informed consent will always be obtained prior to any study specific procedures or investigations, including those necessary to confirm eligibility or arranging any study-specific visits.

The original signed consent form will be retained in the Investigator Site File, with a copy in the relevant clinical notes and a copy provided to the participant. The participant will specifically consent to their GP being informed of their participation in the study. The right to refuse to participate without giving reasons will be respected.

The information sheet and consent form for the study will be available only in English. Interpreters will be arranged for all visits of participants who require them either for verbal translation or for deaf participants wishing to take part in the study, via local NHS arrangements. Qualified interpreters will be used to explain the consent form and information sheet, and great priority will be placed on finding the most direct communication. Participants who lack capacity to consent for themselves will be excluded from the study.

### Intervention

Levothyroxine (LT4) will be used to achieve a target TSH level of 0.4 to 2.5 mU/L.

### Control

A placebo will serve as the control.

#### Randomisation

Participants will be randomised using a computerised randomisation algorithm, stratified by type of MI (NSTEMI versus STEMI), in a 1:1 ratio to levothyroxine therapy or placebo (as container or bottle numbers), starting within 21 (+/− 7) days of the AMI (date of diagnosis or the date of admission to hospital, whichever is later). In addition, investigators will be unaware of allocation and will be blinded to treatment groupings. Randomisation will be administered centrally via Newcastle Clinical Trials Unit using a secure password-protected web-based system.

### Blinding

Assignment to either the LT4 or placebo arm will be blinded to the participant as well as study team (double-blind). Code break envelopes will be available at the pharmacy at each site.

Study medication will be prescribed by the study team at each site using a trial-specific prescription (documenting the required IMP pack number), and dispensed according to local pharmacy practice. Independent study physicians, who will be unblinded, will adjust study drug dose as per protocol. The relevant pharmacy will hold a corresponding list allowing pharmacy staff to correlate IMP pack number with relevant packaged IMP dose for any particular IMP bottle, thus maintaining the double-blind.

At the final visit, the integrity of the blind will be assessed by asking the participants: “It is important for the interpretation of the results of the study that we ask you the following: Do you think you were taking LT4 or placebo? Why do you think this?”

### Study medication

The LT4 medication (IMP) will be used within their licensed indications in this study [29,30]. Nonetheless, since LT4 is being compared to a placebo, additional information is being gathered on the safety and efficacy, and manufacture will involve de-blistering/over-encapsulation; the placebo will be treated as an Investigational Medicinal Product (IMP) for the purpose of this study, and will be labelled and handled accordingly.

The study drug will be sourced, assembled and packaged by Newcastle Specials (Pharmacy Production Unit) at The Newcastle upon Tyne Hospitals NHS Foundation Trust, MIA (IMP) 17136. The LT4 to be used in this study will be generic Levothyroxine 25 mcg and 50 mcg tablets (Amdipharm Mercury Ltd). This will be purchased in blister packs.

The double-blind will be achieved by de-blistering and over-encapsulation, using a capsule filler of Lactose BP. For doses that are multiples of 50 mcg, we will over-encapsulate Levothyroxine 50 mcg tablets; for the remaining 25 mcg, 75 mcg and 125 mcg dose increments, we will over-encapsulate Levothyroxine 25 mcg tablets. This ensures the capsule size is kept as small as possible for swallowing purposes (DB capsules Size A, colour: Swedish orange; manufacturer: Capsugel). Capsules will be re-packaged into an appropriate bottle container (polypropylene) and labelled.

As it is not feasible to predict in advance exactly how much IMP must be packaged per study dose, predictions will be based on known current doses as per routine clinical practice; production run(s) will generate surplus stock per IMP dose. The LT4 medication will have a shelf-life maximum of 24 months (less any reduction in expiry due to the shortest expiry date of blister packs purchased). The IMP should NOT be stored above 25 °C and must be stored in the original container to protect it from light and moisture, as per the clinical trial label. A temperature log will be maintained as per local pharmacy procedures, and appropriate IMP storage will be guaranteed until the IMP arrives in patient possession.

The side effects of LT4 (25 mcg and 50 mcg) are well known and documented [29,30]. The total daily dose provided to each participant will be between 25 mcg and 150 mcg.

### Administration of the study drug

Participants will be randomised to either LT4 or placebo to be taken orally once daily. This will be discussed at the time of consent and will be clear in the patient information sheet. Initial starting dose of LT4 will always be 25 mcg daily. To achieve the desired target TSH levels in the LT4-treated group (target TSH levels 0.4 to 2.5 mU/L), participants will have their TSH levels checked every 4 weeks and concomitant dose of their LT4 altered by 25 mcg daily, if required. The IMP will be provided as 5 week supplies of LT4 or matching placebo (dispensed separately at each visit), packaged into appropriate individual polypropylene bottles. The label on the bottle will not indicate details of the arm of the study to which the participant has been randomised. The label will instead contain a pack number, which will be the link to the relevant dose.

Study medication will be prescribed by the PI or co-investigator at each site using a trial-specific prescription (documenting the required IMP pack number), and dispensed according to local pharmacy practice. Independent study physicians who will be unblinded will adjust study drug dose as per protocol. The relevant pharmacy will hold a corresponding list allowing the pharmacy staff to correlate the IMP pack number with relevant packaged IMP dose for any particular IMP bottle, thus maintaining the double-blind.

Participants will be informed of potential adverse reactions and advised to contact the relevant study team if required. A study-specific participant contact card will also be provided.

Once randomised, the participant will begin their study medication on Day 0.

At 4, 8, 12 and 24 weeks (+/− 7 days), serum TSH levels will be checked and LT4 doses adjusted as follows (the next bottle of IMP should be started at week 4, 8, 12, 24 +/− 7 days, respectively):

LT4 group:

- TSH 0.4 to 2.5 mU/L: Continue LT4 at current dose once daily
- TSH > 2.5 mU/L: LT4 increased by 25 mcg daily
- TSH < 0.4 mU/L: LT4 reduced by 25 mcg, or if already on 25 mcg daily (the lowest possible dose), then recheck TFTs in 4 weeks. If TSH is ≥0.4 mU/L then continue on current dose. Otherwise, if TSH continues to be <0.4 mU/L on the repeat blood test, then the participant will be withdrawn from the study.

Placebo group:

- Individuals in this group will have a fresh bottle of IMP starting at 4, 8, 12, 24 +/− 7 days after blood tests to maintain the double blind.

LT4 treatment based on the above regimen will continue for a total of approximately 52 weeks at which point the final set of study specific assessments will be made (with a further follow-up phone call at approximately 53 weeks). If a participant in the placebo arm has a TSH >10 mU/L then their TFTs will be rechecked in 4 weeks. If, the TSH is ≤10 mU/L on the repeat test then the participant will continue with the study and, if, the TSH continues to be >10 mU/L then the participant will be withdrawn from the study. At the final visit, participants on each arm of the study will stop their IMP and will be referred back to their GP to have their thyroid function checked in 6 to 12 weeks. The GP and the patient will be encouraged to discuss whether there is a need to commence LT4 therapy in these individuals based on the clinical situation. The GP letter (sent once the participant is randomised) and the GP follow-up letter (sent after each participant completes their IMP) outlines the suggestion to recheck TSH levels at 6to 12 weeks for each participant once they have completed their IMP.

At the end of each visit, participants will be asked to return any surplus study drug in the original packaging to the study team, who will verify and document compliance. All unused study medication and packaging will be sent to the local pharmacy for documentation and destruction as per local policy (following appropriate reconciliation by the Trial Manager). Documentation of prescribing, dispensing and return of study medication shall be maintained for study records.

### Data collection and outcome assessments

The schedule of events for ThyrAMI 2 is shown below in Table 1.

**Table 1:** **Schedule of events for ThyrAMI 2**

	**Pre-screening**	**Baseline**			**Randomisation**	**follow-up**							
**VISIT**		**1A**	**1B**	**1C**		**2**	**3**	**4**	**5**	**6A**	**6B**	**6C**	**7**
**assessments**	**Within 24 hours of AMI**	**7 to 10 days post AMI**	**Day 0**			**Week 4 (+/− 7 days)**	**Week 8 (+/− 7 days)**	**Week 12 (+/− 7 days)**	**Week 24 (+/− 7 days)**	**Week 52 (+/− 7 days)**			**Week 53 (+/− 7 days)**
Identification and initial approach (including provision of PIS)	X												
Eligibility criteria checked	X												
Written informed consentǂ		X											
Physical examination (height ****, weight, BP, pulse)		X										X	
Venepuncture		X*				X**	X**	X**	X**			X*	
Clinical history (medical history, medication list)		X											
Questionnaires (SF12, MLWHFQ, CES-D) completed		X										X	
Safety checks (ECG, pulse oximetry and NYHA assessment		X				X	X	X	X			X	
Cardiac MRI			X							X			
EndoPat, Badimon chamber, VerifyNow and TEG analysis				X							X		
Randomisation (after written informed consent obtained) and study drug dispensation					X ***#***								
Study medication prescribed and dispensed (following LT4 dose assessment at visits 2 to 5 only)						X	X	X	X				
Study medication compliance checks						X	X	X	X			X	
Concomitant medication						X	X	X	X			X	
Adverse events						X	X	X	X			X	X
Serious adverse events						X	X	X	X			X	X

X* 20 ml blood: TSH, FT4, FT3, TPO antibodies, Total cholesterol, HDL, Triglycerides, serum BNP, endothelial markers (10 ml gel/gold tube and 10 ml EDTA/purple tube).

X** 5 ml blood: TSH, FT4, FT3 only (gel/gold tube).

Height ****: To be measured at baseline visit only (or could be self-reported if apparatus not available; for example at participant’s home).

ǂ After full discussion and answering any questions.

List of Abbreviations:

BP, blood pressure; BTF, British Thyroid Foundation; CES-D, Centre for Epidemiologic Studies Depression Scale; ECG, electrocardiogram; LT4, levothyroxine; MLWHFQ, Minnesota Living With Heart Failure Questionnaire; MRI, magnetic resonance imaging; NYHA, New York Heart Association; PIS, patient information sheet(s); SF12, short-form health survey; TEG, thromboelastography.

### Data handling and record keeping

Medical information obtained at each visit will be recorded in the subject’s medical notes or other source documentation in real time. Data will be collected and entered into a secure, validated clinical data management system (MACRO), by an authorised member of the research study team. The data management system will be 21 CRF Part 11 compliant. Data for each visit will be entered by relevant local staff at each site. The clinical data management system will be web based; allowing access to authorised staff via password protection. Data will be handled, computerised and stored in accordance with the Data Protection Act 1998. No participant identifiable data will leave the study site (CRFs will identify participants by initials, date of birth and unique patient number only). Strict confidentiality will be ensured while dealing with patient-sensitive data in accordance with the Caldicott Guardian’s recommendations (applications will be made to the relevant Caldicott Guardian for use of NHS patient data).

All study data will be held in strict confidence by the investigators/research team. Data and documents will be stored in locked cupboards. A confidential list of trial identifiers and corresponding patient identifying details will be held at site in a locked cupboard by the Principal Investigator. The quality and retention of study data will be the responsibility of the CI. All study data will be retained in accordance with the latest Directive on GCP (2005/28/EC) and local policy.

### Submission of accrual data to the United Kingdom Clinical Research Network

This study will apply for adoption to the NIHR CRN Portfolio. Accrual data will be submitted on a monthly basis, by a delegated member of staff at the Newcastle Clinical Trials Unit, in accordance with NIHR CRN guidelines.

### Statistical considerations for ThyrAMI 2

#### Sample size

Left ventricular ejection fraction (LVEF) is considered to be the strongest predictor for adverse outcomes after an acute myocardial infarction (AMI). Previous randomised trials in patients with acute myocardial infarction that showed reduced mortality and morbidity demonstrated concurrent absolute differences in LVEF of 3 percentage points or more [31,32]. Therefore, we considered this to be a feasible and clinically important difference. The sample size calculation is based on the assumption that impact of the intervention will be assessed using analysis of covariance and uses the method described by Frison and Pocock (1992) as implemented in the sampsi procedure in Stata 13 [33].

The ThyrAMI 2 study is designed with 90% power to detect an overall difference of 3% in LVEF between the two groups (3% improvement in the placebo group and 6% in the LT4 group) 12 months after AMI, at a two-sided significance level of 5%. The SD of the effect measure is estimated to be a broad 6% [34]. Using ANCOVA (differences in change in EF from baseline between placebo and LT4 taking into account variability in the baseline with correlation between baseline and follow-up of 0.75), we calculate that 47 patients would need to be enrolled in each group, allowing for a 10% drop-out.

### Sampling and recruitment

A feasibility analysis showed that approximately 150 patients per month are admitted to two coronary care units in the northeast. Based on our previous study, we anticipate that 10 to 17% of these patients will have SCH [18]. If 50% of these patients will consent to participate in the trial, we will complete recruitment in 12 months at 8 participants per month.

### Statistical analysis

The primary analysis will be an *intention to treat* analysis of change in LVEF before and after levothyroxine therapy. The dependent variable will be LVEF at follow-up; baseline LVEF will be included as a covariate. Residual errors will be assumed to have a normal error distribution. The difference between patients randomised to LT4 and placebo will be fit as a fixed effect. Model assumptions will be checked, and if necessary, transformations of the dependent variable will be considered. A secondary *per protocol* analysis of change in LVEF before and after levothyroxine therapy will also be performed. Both primary and secondary outcomes will be measured at baseline (prior to start of the interventional medication as well as at 52 weeks (end of study). Change in secondary outcomes between the LT4 and placebo groups will be compared using analysis of covariance. Variables that are not normally distributed will be transformed prior to parametric analyses.

### Compliance and withdrawal

#### Assessment of compliance

Compliance with study medication (IMP) will be assessed and documented by the research team by checking and recording the number of returned capsules at each visit. This allows any issues to be addressed immediately with the participant. Compliance will be classed as good if between 80 to 100%. The local pharmacy will also document all unused study medication/packaging prior to appropriate reconciliation by the Trial Manager.

### Withdrawal of participants

Participants have the right to withdraw from the study at any time for any reason without giving a reason. The investigator also has the right to withdraw patients from the study drug in the event of inter-current illness, adverse events, serious adverse events, suspected unexpected serious adverse reactions, protocol violations, administrative reasons or other reasons.

Should a participant decide to withdraw from the study, their wishes will be respected. These participants will be asked to complete a “confirmation of withdrawal” form to document their decision. However, for participants that withdraw due to reasons other than related to tolerance of MR scanning, they would be offered the chance to have the primary outcome assessment (cardiac MR imaging) performed at the time of withdrawal.

Participants who have serum TSH <0.4 or >10.0 mU/L on two separate occasions at least 4 weeks apart whilst on study drug will be withdrawn from the study due to safety reasons. For study participants who withdraw from the study, a replacement eligible participant will be recruited, if the study is still recruiting.

### Monitoring, quality control and assurance

The study will be managed by the Chief Investigator, Research Nurse and through NCTU. The Trial Management Group (TMG) will include the Chief Investigator, Trial Statistician, Trial Manager and Research Nurse.

The Principal Investigators will be responsible for conducting the study day-to-day at the site.

The Chief Investigator, Research Nurse and NCTU will provide day-to-day support for the sites and provide training through a site initiation visit and routine monitoring visits.

Quality control will be maintained through adherence to Newcastle Biomedicine Clinical Research Platforms SOPs, study protocol, the principles of GCP, research governance and clinical trial regulations.

An independent Data Monitoring and Ethics Committee (DMEC) comprising independent team members: chair, local expert and statistician will be convened to undertake independent review. The purpose of this committee will be primarily to monitor safety endpoints. The committee will meet at least twice a year after recruitment commences to identify any safety issues.

A Trial Steering Committee (TSC) chaired by an independent lay expert will be established to provide overall supervision of the trial. The TSC will meet at least yearly. At the request of the TSC, interim meetings, in person or by teleconference, will be organised. Any major trial issues may need to be dealt with between meetings, by phone or by email.

Monitoring of study conduct and data collected will be performed by a combination of central review and site monitoring visits to ensure the study is conducted in accordance with GCP and with adherence to the Trial Monitoring Plan, which will be agreed on with the Sponsor. Study site monitoring will be risk-based and will be undertaken by NCTU. The main areas of focus will include consent, serious adverse events, essential documents in study files and drug accountability and management.

Site monitoring plan is likely to include the following:

1. All original consent forms will be reviewed. The presence of a copy in the participant hospital notes will be confirmed for a percentage of participants.
2. All original consent forms will be compared against the study participant identification list.
3. All reported serious adverse events will be verified against treatment notes/medical records.
4. The presence of essential documents in the investigator site file and study files will be checked.
5. Source data verification for a selection of data/participants entered into the study (for example, primary endpoint data and eligibility data).
6. Drug accountability and management will be checked.
7. Central monitoring will include:
8. All applications for study authorisations and submissions of progress/safety reports will be reviewed for accuracy and completeness, prior to submission.
9. All documentation essential for study initiation will be reviewed prior to site authorisation.

All monitoring findings will be reported and followed up in a timely manner.

The study may be subject to inspection and audit by designated staff from the Gateshead Health NHS Foundation Trust under their remit as Sponsor, and other regulatory bodies to ensure adherence to GCP. The investigator(s)/institutions will permit trial-related monitoring, audits, REC review and regulatory inspection(s), providing direct access to source data/documents. The study’s Patient Information Sheets and consent forms will make it clear that this level of access to records is required.

### Pharmacovigilance

The safety of LT4 in this study will be evaluated by examining the occurrences of all adverse events, adverse drug reactions, unexpected adverse reactions, serious adverse reactions or suspected unexpected serious adverse reactions as defined by the Medicines for Human Use (Clinical Trials) Regulations [35]. The relationship and expectedness of the adverse event or reaction (causality) will be assessed by the site investigator and relayed to the chief investigator who, on behalf of the sponsor (Gateshead Health NHS Foundation Trust), will notify the regulatory bodies, the Medicines and the Healthcare Products Regulatory Agency (MHRA) and the research ethics committee (REC), within 7 days for fatal and life-threatening events and 15 days for non-life-threatening events. All study investigators will be notified.

Most adverse events and adverse drug reactions that occur in this study, whether they are serious or not, will be expected treatment-related toxicities due to the drugs used in this study. For a full list of expected undesirable effects of Levothyroxine, please refer to the relevant Summaries of Product Characteristics [29,30].

We do not anticipate adverse effects of LT4. Nevertheless, safety of participants is of paramount importance, and therefore, LT4 dose will be commenced at a small dose (25 mcg daily) and the dose increased every 4 weeks by 25 mcg daily until the serum TSH is within the required range. Safety measures (ECG, symptoms of heart failure, and pulse oximetry) will be assessed at visits 2 to 6. If severe hypothyroidism develops (TSH >10 mU/L with low FT4 levels), then symptoms including fatigue, weight gain and poor memory may develop. For such individuals, we will recheck TFT in 7 days. If serum TSH remains above 10 mU/L, then such patients will be withdrawn from the study. In addition, we will assess the health of participants via a health status questionnaire. Participants will also be provided with a helpline number in case they experience any problems.

For purposes of this protocol:

1. All non-serious adverse events will be recorded at the follow-up visits 2 to 6.
2. Any serious adverse events will be recorded throughout the duration of the trial until week 52 (+/− 3 days).
3. Serious adverse events exclude any pre-planned hospitalisations (for example, elective surgery) not associated with clinical deterioration.
4. Serious adverse events exclude routine treatment or monitoring of the studied indication (that is, hypothyroidism), not associated with any deterioration in condition.
5. Serious adverse events exclude elective or scheduled treatment for pre-existing conditions that did not worsen during the study.

### Confidentiality

Personal data will be regarded as strictly confidential. To preserve anonymity, any data leaving the site will identify participants by their initials, date of birth and a unique patient number only. The study will comply with the Data Protection Act, 1998, and Caldicott guidelines. All study records and Investigator Site File(s) will be kept at site in a locked filing cabinet with restricted access. The Trial Master File will be held at Newcastle Clinical Trials Unit in a locked filing cabinet with restricted access.

All laboratory samples (sent for analysis to the Department of Clinical Biochemistry, Gateshead Health NHS Foundation Trust) will be labelled with a unique patient number, initials and date of birth (linked in anonymised form), with the centre number and visit number and date/time of sample.

### Ethics and regulatory issues

The conduct of this study will be in accordance with the recommendations for physicians involved in research on human subjects adopted by the 18th World Medical Assembly, Helsinki 1964 and later revisions.

Favourable ethical opinion from an appropriate Research Ethics Committee (NRES Committee North East - Sunderland, 14/NE/0151) and Clinical Trial Authorisation from the Medicines and Healthcare Product Regulatory Agency has already been obtained. The approving R&D committees include the following: The Newcastle upon Tyne Hospitals NHS Foundation Trust, Gateshead Health NHS Foundation Trust, City Hospitals Sunderland NHS Foundation Trust and Northumbria Healthcare NHS Foundation Trust. All local approvals will be obtained before recruitment commences at each site. The Study Coordination Centre will require a written copy of local approval documentation before initiating each centre and accepting participants into the study.

## Discussion

Acute myocardial infarction (AMI) and its related complications represent a major public health problem. Despite improvements in reperfusion therapy, the 2020 World Health Organisation projections view the high incidence of post-ischaemic heart failure as the most important cause of morbidity and mortality [36]. Limitation of infarct size extent and recovery of dysfunctional contractile segments are the key elements for preventing post-ischaemic left ventricular remodelling. Therefore, despite research into new reperfusion strategies, a new perspective involves studying mechanisms by which myocytes can self-protect at times of stress, which can prevent irreversible cell death. In this regard, thyroid hormones are increasingly being recognised as significant players in the pathogenesis and the recovery and repair period of AMI [36,37].

Even mild hypothyroidism, known as subclinical hypothyroidism, is associated with worse cardiovascular outcomes. The prevalence of SCH is higher than that of type 2 diabetes mellitus (5-10%) and is more common in women and the elderly [4,38]. Small intervention trials of levothyroxine in SCH have shown improvement in left ventricular function, vascular endothelial function and atherogenic lipid particles [8]. The Cardiovascular Health Study showed that SCH participants who were treated with levothyroxine had a 72% reduction in heart failure events [14]. Thus, even mild hypothyroidism following AMI could be an important marker for poor outcome. No clinical trial has assessed outcomes of SCH in myocardial infarction and whether treating this state can improve outcomes by increasing left ventricular function, reducing thrombogenesis, improving endothelial function and quality of life. Therefore ThyrAMI 1 and ThyrAMI 2 will be the first trials investigating SCH in myocardial infarction to give a better insight into whether thyroid hormone levels are a key target for improving cardiovascular outcomes.

## Trial status

The study received National Health Service (NHS) ethics approval on 28 May 2014 (NRES Committee North East - Sunderland), MHRA approval on 30 October 2014, and NHS Trust R&D governance approvals are awaited. Recruitment will commence on 22 December 2014 and proceed until May 2016. Results will be submitted for publication in 2017.

## Abbreviations

ADR: adverse drug reaction
AMI: acute myocardial infarction
BP: blood pressure
BTF: British Thyroid Foundation
CES-D: Centre for Epidemiologic Studies Depression Scale
CI: chief investigator
CRF: clinical research facility
CRF: clinical research form
CRN: clinical research network
CTA: clinical trial authorisation
ctIMP: clinical trial of an investigational medicinal product
DMEC: data monitoring and ethics committee
ECG: electrocardiogram
FT4: free thyroxine
FT3: free triiodothyronine
GCP: good clinical practice
GP: general practitioner
HDL: high-density lipoprotein
HTA: health technology assessment
IMP: investigational medicinal product
LT4: levothyroxine
LV: left ventricular, MHRA, medicines and healthcare products regulatory agency
MLWHFQ: Minnesota Living With Heart Failure Questionnaire
MRI: magnetic resonance imaging
NIHR: National Institute for Health Research
NYHA: New York Heart Association
PI: principal investigator
PIS: patient information sheet(s)
QoL: quality of life
R&D: research and development
RCT: randomised controlled trial
REC: research ethics committee
SCH: subclinical hypothyroidism
SF12: short-form health survey
SOP: standard operating procedure
TEG: thromboelastography
TMG: trial management group
TPO: thyroid peroxidase antibody
TSC: trial steering committee
TSH: thyroid stimulating hormone
